# Multi-Barley Seed Detection Using iPhone Images and YOLOv5 Model

**DOI:** 10.3390/foods11213531

**Published:** 2022-11-06

**Authors:** Yaying Shi, Jiayi Li, Zeyun Yu, Yin Li, Yangpingqing Hu, Lushen Wu

**Affiliations:** 1 School of Mechatronic Engineering, Nanchang University, Nanchang 330047, China; 2 Department of Electrical Engineering and Computer Science, University of Wisconsin-Milwaukee, Milwaukee, WI 53201, USA; 3 Malteurop Malting Company, Milwaukee, WI 53215, USA

**Keywords:** barley seed detection, deep learning, object detection, Yolov5

## Abstract

As a raw material for beer, barley seeds play a critical role in producing beers with various flavors. Unexcepted mixed varieties of barley seeds make malt quality uncontrollable and can even destroy beer flavors. To ensure the quality and flavor of malts and beers, beer brewers will strictly check the appropriate varieties of barley seeds during the malting process. There are wide varieties of barley seeds with small sizes and similar features. Professionals can visually distinguish these varieties, which can be tedious and time-consuming and have high misjudgment rates. However, biological testing requires professional equipment, reagents, and laboratories, which are expensive. This study aims to build an automatic artificial intelligence detection method to achieve high performance in multi-barley seed datasets. There are nine varieties of barley seeds (CDC Copeland, AC Metcalfe, Hockett, Scarlett, Expedition, AAC Synergy, Celebration, Legacy, and Tradition). We captured images of these original barley seeds using an iPhone 11 Pro. This study used two mixed datasets, including a single-barley seed dataset and a multi-barley seed dataset, to improve the detection accuracy of multi-barley seeds. The multi-barley seed dataset had random amounts and varieties of barley seeds in each image. The single-barley seed dataset had one barley seed in each image. Data augmentation can reduce overfitting and maximize model performance and accuracy. Multi-variety barley seed recognition deploys an efficient data augmentation method to effectively expand the barley dataset. After adjusting the hyperparameters of the networks and analyzing and augmenting the datasets, the YOLOv5 series network was the most effective in training the two barley seed datasets and achieved the highest performance. The YOLOv5x6 network achieved the second highest performance. The mAP (mean Average Precision) of the trained YOLOv5x6 was 97.5%; precision was 98.4%; recall was 98.1%; the average speed of image detection reached 0.024 s. YOLOv5x6 only trained the multi-barley seed dataset; the trained performance was greater than that of the YOLOv5 series. The two datasets had 39.5% higher precision, 27.1% higher recall, and 40.1% higher mAP than when just using the original multi-barley seed dataset. The multi-barley seed detection results showed high performance, robustness, and speed. Therefore, malting and brewing industries can assess the original barley seed quality with the assistance of fast, intelligent, and detected multi-barley seed images.

## 1. Introduction

Modern agriculture industries have been well developed using agricultural science, and the modern malting industry urgently needs intelligence assistance. The image processing method is widely used in agriculture as a form of touchless and automatic detection, and is a promising method for barley recognition. There are about 140 varieties of barley in the United States and Canada [1]. According to the growing environment and proficiency, farmers choose the correct barley variety to grow. Barley seed is a primary material for the manufacturing of beer. Generally, specific types of beer are made with malt from specific barley seeds; different malts make different beer flavors. Malts with unexpected mixed barley seed varieties can result in poor modification and uniformity, reduced extractives, elevated beta-glucans and arabinoxylans, and poor flavors in beer products [2,3]. These may lead to reduced malt yields, increased barley seed costs, and potential economic losses for malting businesses. With continuous improvement at the brewing level and the pursuit of beer quality, research on beer products is urgently required [4]. Identifying barley seed varieties is the first crucial step in ensuring the purity of beer flavors.

**Figure 1:**
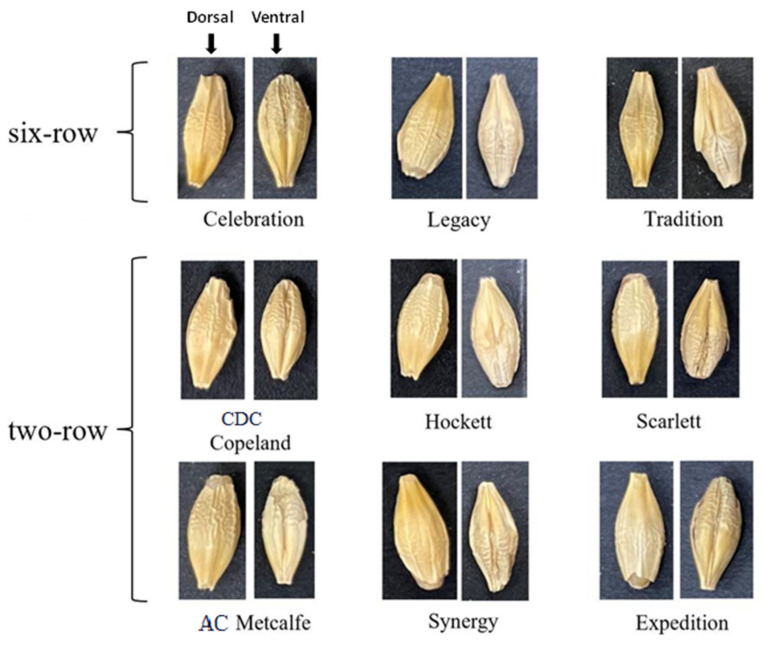
Images of nine barley varieties.

As shown in Figure 1, there are nine varieties of barley seeds. Barley seeds are about 6–8 mm long and 3–4 mm wide, and have a dorsal side without creases (left side image in every type of barley seed) and a ventral side with creases (right side image in every type of barley seed). Different barley seeds have many different features on the dorsal and ventral sides. They show significant differences in barley kernel shape and color, morphology at the base of the lemma, and the density of spikelets [5]. These different features are related to many factors, such as the growing environment (weather, soil condition, planting season), cultivating conditions (water composition, fertilization), and the spikelet location (two-row and six-row barley). However, the same barley seeds also have minor morphology variations because of these different cultivating factors. Therefore, barley seeds, by using the ventral and dorsal sides, have many standardizable features for detection. Indeed, professionals judge barley seed varieties by these different features. However, they are difficult to directly identify with the naked eye, and the varieties are small in size and have similar features; thus, the manual detection of barley seeds can result in detection errors. Barley seed variety identification usually uses morphological analysis, immunological analysis, protein electrophoresis, and DNA fingerprinting technology. The morphological analysis method, also called visual analysis, uses touchless technology and does not destroy the structure of the seeds. Other methods can achieve high identifying accuracy, but destroy the structures of the seed samples in the process; they are also highly time-consuming and high in cost, and can require professional laboratories.

Intelligent detection technology is widely used in image detection, such as single-object images, multi-object images, small object images, and sub-category images [6]. Machine learning includes supervised learning, unsupervised learning, and reinforcement learning. Image recognition is mainly processed by supervised learning. When using supervised learning technology, the target objects are manually labeled by the user, and these labeled images are then trained to obtain the corresponding model. The model can test unlabeled images to identify the target object [7]. When using intelligent detection technology, the methods used evaluate barley seed variety and quality at high speeds, low costs, and with high accuracy. Artificial intelligence and supervised deep learning networks help with barley seed detection and recognition. This study aims to build an efficient, automatic artificial intelligence detection method based on a multi-barley seed dataset with random varieties and numbers in each image. Notably, there are no published barley seed detection studies on multi-barley seed datasets. The intelligent detection method sets professionals free from visual inspection, and is a helpful method by which to check the purity of barley seeds. Professionals could use the barley variety detection images to judge if the collected malt barley seeds are qualified for making malts and beers.

## 2. Object Detection Methods

Traditional object detection includes three processes: acquiring detection bounding boxes, extracting interest features of the objects, and training the classifier [8,9,10]. This method achieves high detection accuracy, but is time-consuming, has high window redundancy, and uses an untargeted detection process. With the development of computer technology and hardware facilities, object detection algorithms based on deep learning networks are widely used and have high performance. Object detection based on deep learning networks is divided into two-stage and one-stage detection.

**Figure 2:**
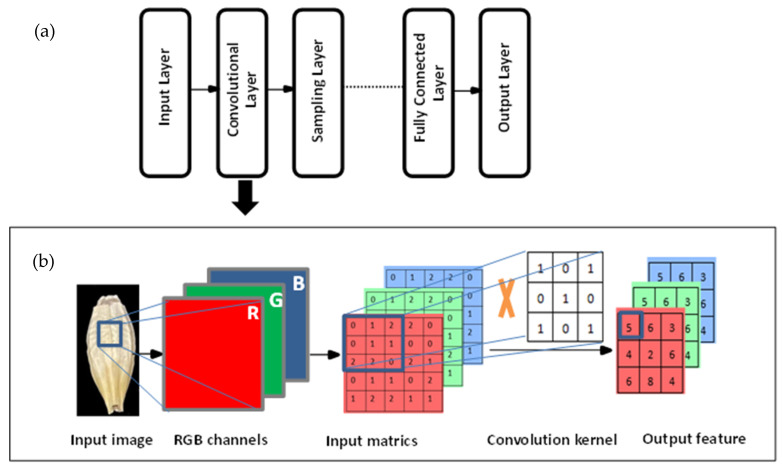
Building blocks of a Convolutional Neural Network (**a**) and explanation of feature extraction (**b**).

**Figure 3:**
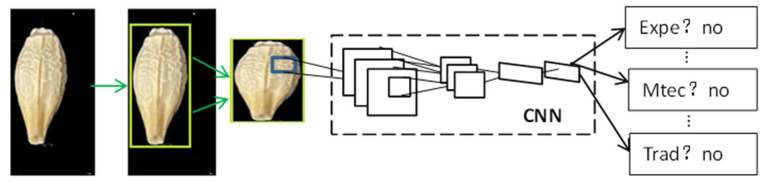
Network architecture of R-CNN.

Two-stage methods, also called region-proposal-based methods, use sliding windows to detect the objects in the image and then use a Convolutional Neural Network (CNN) to recognize detected objects [11]. CNN is a representational algorithm for deep learning networks. As shown in Figure 2, a basic CNN structure includes a convolutional layer, a ReLU layer, a pooling layer, and a fully connected layer [12]. The convolutional layer with filters and the ReLU layer with ReLU activate function transfer the input image information into invariant feature information, such as color, shade, and outline. The pooling layer can contract the size of the image and retain the critical information in the images and in the fully connected layer output images. CNN can automatically extract features and efficiently enhance the direct learning of image features based on processing high dimension datasets. In 2014, Girshick et al. proposed the R-CNN network of two-stage detection methods [13]. As shown in Figure 3, R-CNN uses selective search algorithms to extract all object region proposals and features from region proposals by CNN. Finally, these features input the SVM classifier by conducting bounding box regression to classify region proposals. The R-CNN algorithm significantly improves object detection performance in comparison to traditional algorithms. However, feature extraction for each region proposal takes a long time; many image feature files take up considerable space, and the detection speed is often too slow. The R-CNN algorithm represents the classical two-stage method. Although Fast R-CNN [14], Faster R-CNN [15], Mask R-CNN [16], and other two-stage algorithms perform well, the detection speed is still too low to satisfy the requirements of some scenarios.

**Figure 4:**
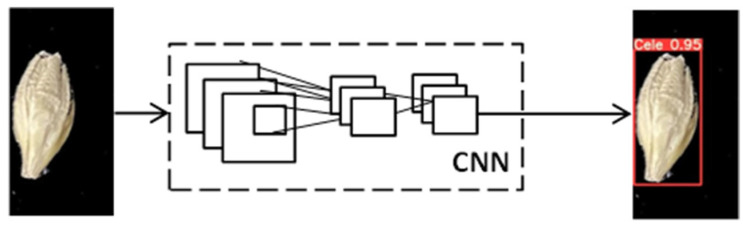
YOLO detection system.

**Figure 5:**
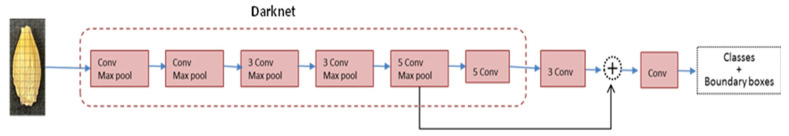
Network architecture of YOLOv2.

**Figure 6:**
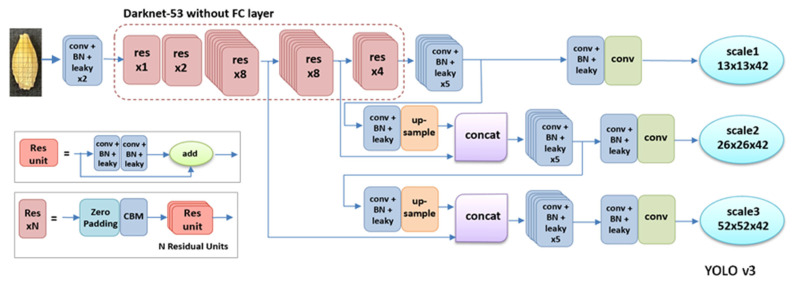
Network architecture of YOLOv3.

**Figure 7:**
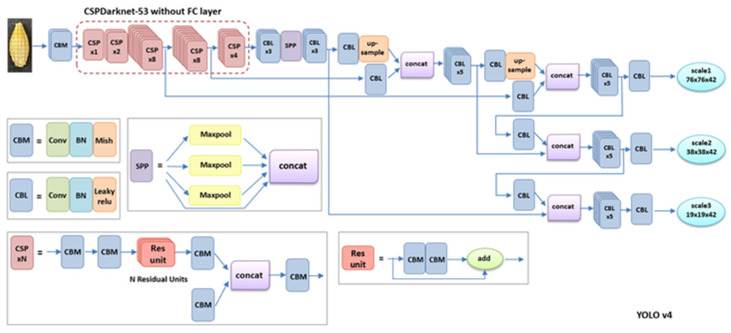
Network architecture of YOLOv4.

**Figure 8:**
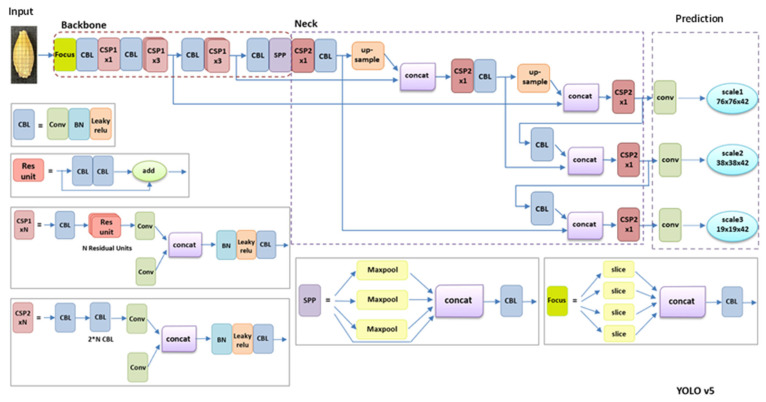
Network architecture of YOLOv5.

In 2016, Redmon et al. proposed the YOLO algorithm as a one-stage method, as shown in Figure 4 [17]. Compared with a two-stage detection method, the YOLO series algorithms, also referred to as regression or classification-based methods, extract features directly from the networks to predict both object classification and localization [17]. Moreover, the speed of YOLO algorithms is greatly improved and the accuracy rate remains high. The one-stage algorithms are widely used at fast speeds and with high accuracy. In 2017, Redmon and Farhadi proposed YOLOv2, a new training method that directly predicts the offset based on the grid and the anchor-replaced bounding box to obtain a stable training process [18]. Figure 5 displays the YOLOv2 detection structure. YOLOv2 uses Darknet-19 as its pre-trained network and adds the Batch Normalization layer for higher performance. However, YOLOv2 was not overly effective for small object detection. In 2018, Redmon and Farhadi proposed YOLOv3, which uses a more complicated framework, Darknet-53, and adds FPN after removing softmax as a selective classification [19]. FPN uses nine anchor boxes, including three small, three medium, and three big boxes [20]. Thus, YOLOv3 achieves higher speed and accuracy, as shown in Figure 6. An improved version of YOLOv3, YOLOv4, was proposed in 2020 [21]. YOLOv4 uses multi-anchors for single-ground truth, while YOLOv3 uses a single anchor for single-ground truth, as shown in Figure 7. YOLOv4 also uses the CIoU_loss function and mosaic data augmentation to perform more effectively [21]. YOLOv5 has been more recently proposed, and its structure is similar to YOLOv4; using an adaptive anchor based on the different labeled datasets. YOLOv5 series networks have the advantages of having a small size, fast speeds, and high accuracy, as shown in Figure 8 [22].

## 3. Objectives of the Study

Object detection tasks include classification and localization, which belong to the multi-task process. An automatic intelligent imaging processing detection method for mixed barley variety identification would replace traditional manual methods used in the agricultural and food processing industries. There is no real need for professionals to manually classify these barley varieties; machines could automatically judge these varieties. This study aims to automatically identify all barley seeds in the images when inputting captured barley seed images into the final well-trained artificial intelligent model. Professionals could use the identified barley seed results to assess if the batches of barley seeds were qualified for malt and beer products.

This study aims to detect nine varieties of barley seeds, featured in random amounts and varieties in each image. Based on the wide varieties of the small and similar features of barley seeds, this is a challenging task. There have been no published studies on the detection of nine varieties of barley seed. In 2019, Kozlowski et al. used a customized model to classify six barley seed varieties and achieved an excellent test accuracy of 93% [23]. In 2018, Dolata and Reiner classified eight varieties of barley seeds based on a viewpoint-aware approach, and achieved a best test accuracy of 88.97% [24]. In 2022, Yaying et al. used the InceptionV3 network to classify nine categories of barley seeds, and achieved a test accuracy of 95.7% [25]. These studies achieved high accuracy for barley seed classification, but they did not detect multiclass barley in one image. Multi-barley seed detection is essential for professionals to directly classify wide varieties of barley seeds and assist professionals in assessing the quality of barley seeds. This will reduce the loss of interest and time consumption in the malting and brewing industries. There has been no published study on the detection of nine varieties of barley seeds. Our study aims to build an automatic well-trained YOLOv5 network to efficiently detect nine varieties of barley seeds and to quickly distinguish multi-barley seeds in one image.

## 4. Materials and Methods

### 4.1. Barley Material

Generally, barley seeds are processed into malt to produce various products, such as beer, some beverages, and various food products. Different varieties of barley seeds have different components and contents, so the malting effect on production differs [26]. Barley (*Hordeum vulgare* L.) samples were taken from large commercial barley farms during the 2018 and 2019 crop years. Barley seeds are divided into 2-row varieties and 6-row varieties. Figure 1 shows nine barley varieties; the 2-row varieties (CDC Copeland, AC Metcalfe, Hockett, Scarlett, Expedition, AAC Synergy) are symmetrical, and the 6-row varieties (Celebration, Legacy, Tradition) are not all symmetrical based on kernel shape. All representative broad-malt varieties were listed by the American Malting Barley Association (AMBA) as recommended malting barley varieties to U.S. growers in 2019 and 2020 [25].

### 4.2. Dataset

**Figure 9:**
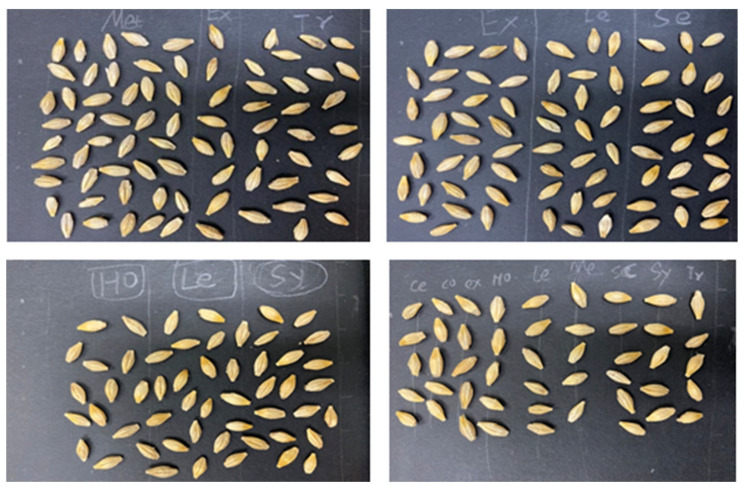
Multi-barley seeds in one image.

**Figure 10:**
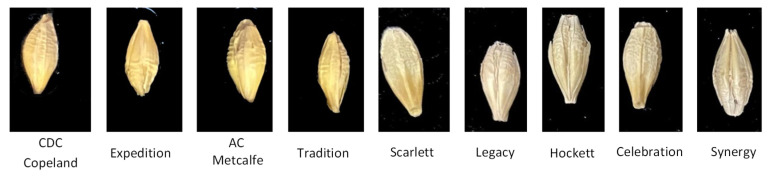
Single-barley seed in one image.

**Figure 11:**
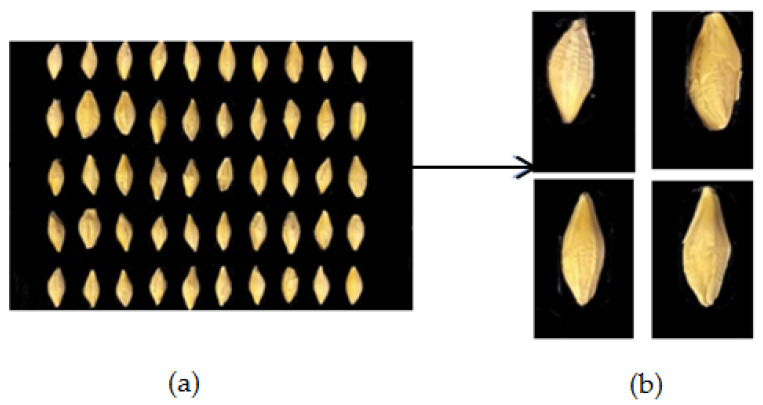
Kernels pre-processing procedure. (**a**) Original image. (**b**) Segmented kernels.

The dataset included nine varieties of barley seed samples. These barley seed images were captured by iOS smartphone iPhone 11 Pro with a 12-mega pixel camera, which is produced by the Apple Computer, Inc in the USA. The barley seeds were small, and there were around 10–90 barley seeds in one photo. Barley seeds were placed on black paper as a background to reduce complex background effects. This study used two types of datasets. One was the multi-barley seed dataset. The multi-barley seed dataset had random amounts and varieties of barley seeds in each image with different angle postures, as shown in Figure 9; we captured 235 photos as multi-barley seed images. The other was the single-barley dataset, collecting the same single-barley seed image for each variety, as shown in Figure 10. The single-barley seed image dataset was photographed initially with 40–50 individual barley seeds uniformly distributed, as shown in Figure 11a. The photographed barley seeds images were segmented into the individual barley seeds of each image, as shown in Figure 11b; we captured 27 photos of the nine varieties of barley seeds, and segmented them into 1080 single-barley seed images. The seed direction was vertical in the single-barley seed images, but multi-barley seeds were randomly placed at different angles. All datasets were photographed on black background paper without blocking each other. The sides of the kernels in the two types of datasets were arbitrary, with both dorsal side kernels and ventral side kernels used. We combined the two types of datasets, the multi-barley seed dataset and the single-barley seed dataset, to achieve high performance of multi-variety barley seed recognition.

### 4.3. Image Pre-Processing

Image pre-processing mainly converts the original photographed dataset into the model training format. Our main pre-processing steps were split into three: individual barley seed segmentation, data labeling, and data augmentation.

**Figure 12:**
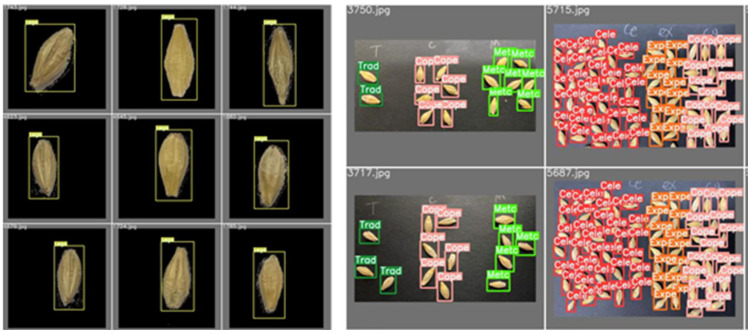
Some examples of labeled images.

**Figure 13:**
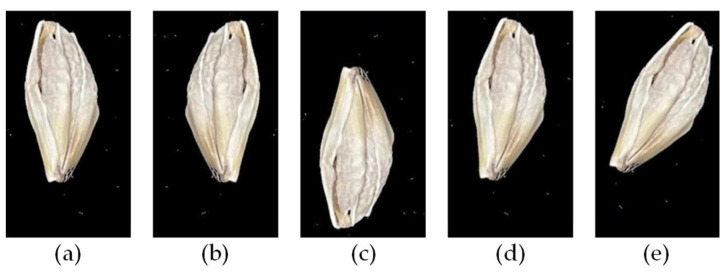
Single-barley image augmentation: (**a**) original image; (**b**) fliph; (**c**) flipv; (**d**) rotation 10°; (**e**) rotation 30°. Note: “fliph” and “flipv” represent flipping the image horizontally and vertically.

**Figure 14:**
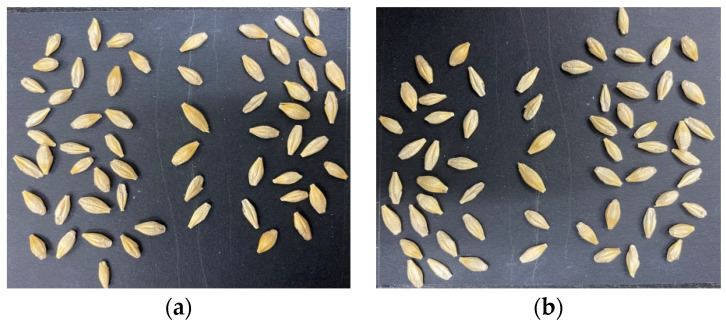

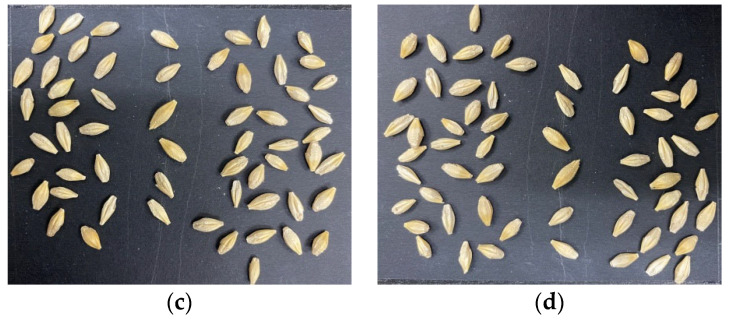
Multi-barley seed image augmentation: (**a**) original image; (**b**) fliph_flipv; (**c**) fliph; (**d**) flipv. Note: “fliph” and “flipv” represent flipping the image horizontally and vertically.

The single-barley dataset was isolated to individual seed kernel regions, as shown in Figure 11. All captured barley images included 40–50 individual seed kernels arranged in order. An image segmentation algorithm located each barley kernel and separated it into individual barley seed images with about 350 × 650 pixels [25]. There were two types of original datasets, including 235 mixed multi-barley seed images and 1080 single-barley seed images. All these datasets were labeled in XML format files, as shown in Figure 12. Generally, image data augmentation expands the diversity of training samples to improve training performance. Basic augmentation methods include noise addition (blur, mosaic) and transformation methods (translation, zoom, flips, shearing, mirror, rotation, color-shifting) [27]. The datasets were labeled, and data augmentation was used on both images and label files. Different dataset types had different statements, and different data augmentation methods were used on different datasets. The single-barley seed images were generally in a vertical statement. Multiple data augmentations were conducted to show various states of random placement in the dataset, such as flipping, rotating small angles, and other data augmentation effects, as shown in Figure 13. Finally, the total number of images was 5400. The images of the multi-barley seed were in arbitrary angle directions. Therefore, we performed classical augmentation to expand the multi-barley seed dataset by flipping, as shown in Figure 14. After data augmentation, the total number of images was 940. Finally, simultaneous data augmentation was performed on all data, including pictures of single-barley seed, multi-barley seed datasets, and the marked content, to obtain more seed state data and increase the dataset.

For the training model, the single-barley seeds dataset and the multi-seed dataset were each split into two separate disjoint sets: the training set and test set. There were 6340 images, with 5400 images for the single-barley seed dataset and 940 images for the multi-barley seed dataset. We split the two datasets by a ratio of 80/20. The training set included 4320 single-barley seed images and 752 multi-barley seed images; providing a total of 5072 images. The test set included 1080 individual barley seed images and 188 multi-barley seed images; totaling 1268 images. After labeling the dataset, we acquired the labeled complex XML format files, mainly including the names of the barley seed varieties and the four maximum and minimum values (xmin, ymin, xmax, and ymax), representing bounding box locations. Normalization linearly transformed the name of the barley seed varieties into zero-indexed variety numbers (starting at 0) and four values of the XML format dataset into the [0,1] range (x_center, y_center, width, and height) of the TXT data format.

### 4.4. Object Detection Models

**Figure 15:**
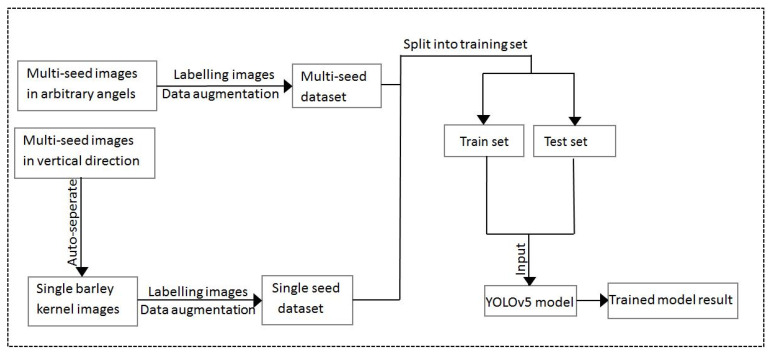
The whole process of barley dataset training.

Figure 15 displays the barley detection process and includes data pre-processing, dataset labeling, data augmentation, dataset split, and dataset training. We used YOLOv5 series networks to train and analyze the dataset, and YOLOv5 improved some structures based on YOLOv3 and YOLOv4. This included input, backbone, neck, and prediction, as shown in Figure 8. After analyzing the features and functions of the barley seed dataset, an effective data augmentation method was used to obtain more valuable training data, and a transfer learning method was used to improve detection accuracy. Since most data or tasks are related, transfer learning will share the parameters of a well-trained model to accelerate and optimize the training efficiency of the new model [28]. YOLOv5 series networks include the transfer learning method, directly improving the training effect. Finally, the processed barley dataset was trained using YOLOv5 series models.

Firstly, the Input includes zooming in on the picture, adopting the mosaic data enhancement method, and automatically calculating the best anchor frame value of the dataset. Then, the backbone structure contains the Focus structure and CSPNet structure. The Focus structure is new in the YOLOv5 network. Its primary function is the slicing operation, which can reduce the number of layers, parameters, amount of calculations, and the usage memory of Cuda, as well as improve the speed of inference and gradient back-propagation. The CSPNet structure is taken from the YOLOv4 network. YOLOv5 combines bottleneck and CSPNet structures to enforce the learning performance of CNN, reduce memory cost, and reduce calculation cost; the neck contains FPN and PAN structures. FPN (Feature Pyramid Network) has nine anchor boxes and strengthens the feature expression of the shallow feature map through the fusion of the upsampling process and the shallow feature map [20]. PANet (Path Aggregation network) aggregates structured shallow features through bottom-up and upward paths, forming a full fusion of different image features and facilitating the transfer of information. Finally, the prediction uses the GIOU_Loss function to evaluate detection performance [29].

YOLOv5 series networks include YOLOv5s, YOLOv5, YOLOv5l, and YOLOv5x models. These models have the same backbone, neck, and head; the only differences are the set depth and width in these models, which decide the depth of models and the number of convolutional kernels. This study used the improved parameters of the YOLOv5 series models to train barley datasets. The trained YOLOv5x6 (Version 6.0 of the YOLOv5x) network achieved the best performance and realized automatic classification and detection.

### 4.5. Loss Function

YOLO series loss calculation is based on objectness, class probability, and bounding box regression. YOLOv5 deploys the BCEclsloss (Binary Cross-Entropy loss) function to calculate the loss of class probability and target score, and the GIOU_Loss (Generalized Intersection over Union loss) as the loss function of the bounding box [29]. In Equation (1), where ${\hat{y}}^{\left(i\right)}$ is the ith scalar value in the model output, $y^{\left(i\right)}$ is the corresponding target value, and ***N*** is the number of scalar values in the model output [30]. Rezatofighi proposed the GIOU_Loss function, which sets the Generalized Intersection over Union (GIOU) loss for bounding box regression [29]. In Equation (2), ***A*** and ***B*** represent the predicted bounding box and the ground-truth bounding box. The Intersection over Union (IOU) requires comparing the similarity between ***A*** and ***B***. ***C*** is the minor enclosing shape, which includes the whole boxes of ***A*** and ***B***. This ratio $\frac{\left|C\backslash \left(A\cup B\right)\right|}{\left|C\right|}$ represents a normalized measure focusing on the empty area between *A* and *B*. Finally, the ***GIOU*** results from ***IOU*** subtracting the ratio in the function, and the GIOU_Loss equals one subtracting the ***GIOU*** in the Equation (3).

(1) $$L=-\frac{1}{N}\overset{N}{\underset{i=1}{\sum }}y^{\left(i\right)}\;log{\hat{y}}^{\left(i\right)}+\left(1-y^{\left(i\right)}\right)log\left(1-{\hat{y}}^{\left(i\right)}\right)$$

(2) $$GIOU=IOU-\frac{\left|C\backslash \left(A\cup B\right)\right|}{\left|C\right|}=\frac{\left|A\cap B\right|}{\left|A\cup B\right|}-\frac{\left|C\backslash \left(A\cup B\right)\right|}{\left|C\right|}$$

(3) $$L_{GIOU}=1-GIOU$$

### 4.6. Assessment Method

Three evaluation parameters [30] were employed in the project:

(1) Precision: The ratio of correctly predicted positive values to total values;
(2) Recall: The percentage of correctly predicted positive values to all the values in the relevant class;
(3) mAP: The average mean value of all categories AP.

Each of the metrics are defined below:

$$\mathrm{Recall}=\frac{\mathrm{True}\;\mathrm{Positive}}{\mathrm{True}\;\mathrm{Positive}+\mathrm{False}\;\mathrm{Negative}}$$

$$\mathrm{Recall}=\frac{\mathrm{True}\;\mathrm{Positive}}{\mathrm{True}\;\mathrm{Positive}+\mathrm{False}\;\mathrm{Negative}}$$

$$\mathrm{mAP}\;=\frac{1}{K}\overset{K}{\underset{i=1}{\sum }}AP_{i}$$

In multi-object detection tasks, the TP (true positive) means the predicted correct box that includes comparing the value of classification and bounding box, which also means the corresponding calculated IOU is over the set threshold value of IOU. Therefore, the FP (false positive) means the corresponding calculated IOU is less than the set threshold value of IOU. False-negative means negative samples are calculated as positive samples. The precision metric is calculated from a true and false positive, and the recall metric is calculated from the true positive and false negative. The mAP value is based on the Precision-Recall curve. The AP is the area of the Precision-Recall curve, also called average precision. Finally, the mAP is the average value of all classes of AP.

## 5. Experiment and Discussion

### 5.1. Model Training

Table 1 displays the configuration of the experimental environment. We used RTX 3090 as the central processor, and the network model was based on PyTorch architecture. We trained the YOLOv5 model on labeled datasets. During training, the hyperparameters of the YOLOv5 network were adjusted to minimize the training loss. The learning rate was set to 0.001, and the batch size was 12. We obtained a well-trained model based on the dataset for 300 epochs. YOLOv5 series networks with different depths and widths trained the mixed datasets to compare their performance. Moreover, the YOLOv5x6 model also trained the dataset with 940 multi-barley seed images. The dataset was split into a ratio of 80/20; the training set had 752 images, and the test set had 188 images. Finally, the split multi-barley seed dataset was trained by the YOLOv5x6 network.

### 5.2. Result Analysis

After model training, we obtained a well-trained model and good performance on barley datasets. We used the YOLOv5 series network to train the dataset and adjusted the hyperparameters of the YOLOv5 network to obtain the corresponding well-trained results shown in Table 2. Compared with other YOLOv5 series, YOLOv5x6 achieved the highest performance and accuracy; trained precision was 98.4%, recall accuracy was 98.1%, and the mAP was 97.5%. Compared with the YOLOv5s trained model, YOLOv5x6 improved the precision by 11.8%, the recall by 19.7%, and the mAP by 20.2%. However, the training time of YOLOV5x6 was 12.48 h longer than the YOLOv5s. Therefore, the network with greater depth and width achieved higher accuracy, but had the longer training time. We also trained the YOLOv5x6 network on the multi-barley seed dataset; trained precision reached 58.9%, recall accuracy reached 71%, and the mAP reached 57.4%. The two mixed datasets achieved 39.5% higher precision, 27.1% higher recall, and 40.1% higher mAP. The single-barley dataset provided more barley seed features, and the dataset that used data augmentation provided more barley postures. These types of single-barley seed images enhanced multi-variety barley seed image detection. Finally, YOLOv5x6 with mixed datasets was the best-trained model and achieved the highest performance; the model detected the inputting of multi-barley seeds images to achieve high accuracy and obtained minor errors. The training time of YOLOv5x6 was 12.48 h, but the well-trained YOLOv5x6 detected one image at an average speed of 0.024 s.

**Figure 16:**
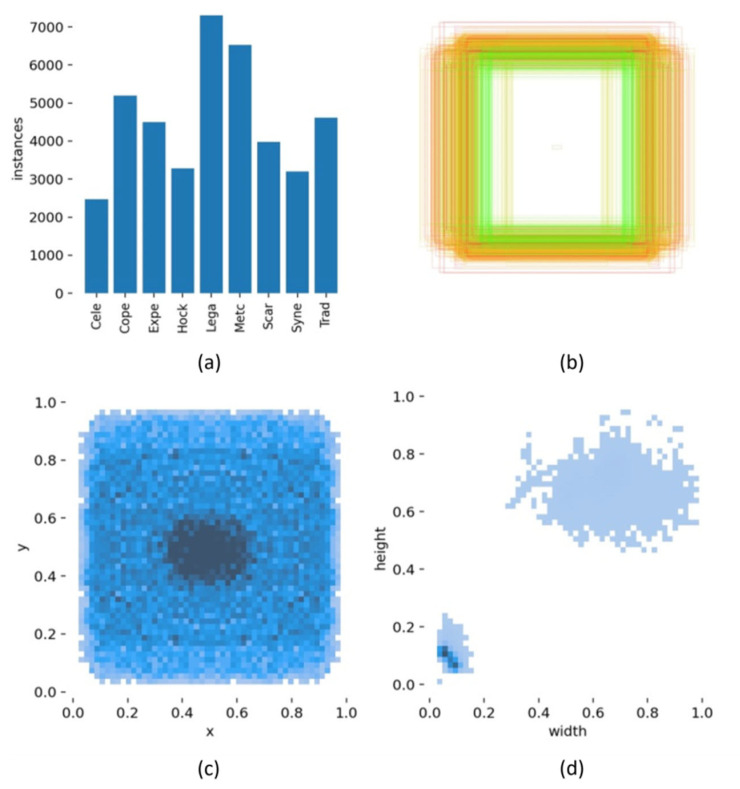
Distribution image of barley seeds varieties and labels: (**a**) the number of each barley seed variety; (**b**) all the normalized labeled boxes; (**c**) all center coordinates for normalized boxes; (**d**) the width and height for all normalized boxes.

**Figure 17:**
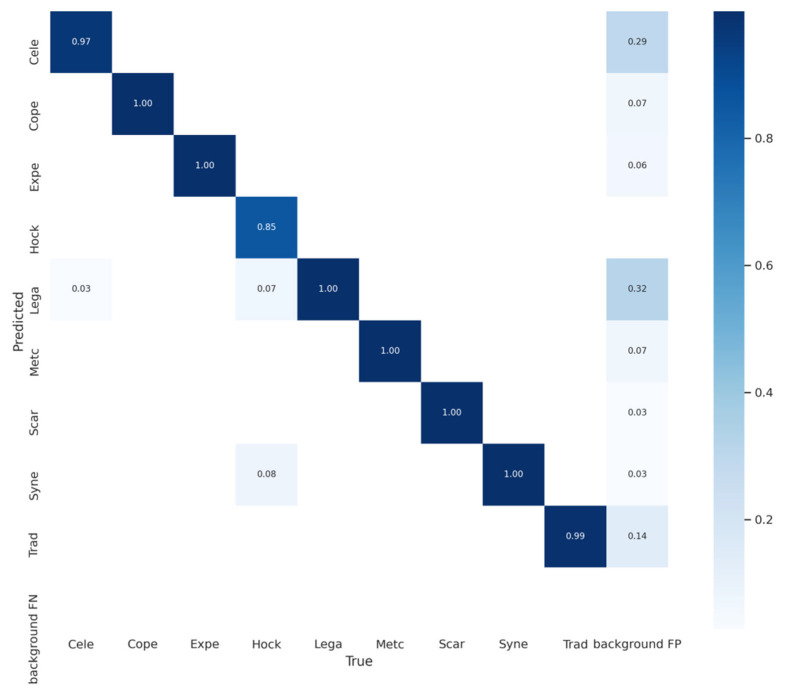
Confusion Matrix of barley seeds detection.

Figure 16 visualizes information for all the barley seeds, including quantity and labeled normalized TXT content, which helped to analyze the YOLOv5x6 model performance. Figure 16a shows the number of each barley seed variety; Figure 16b displays all the normalized labeled boxes; Figure 16c displays all center coordinates for normalized boxes; Figure 16d displays the width and height for all normalized boxes; Figure 17 displays the confusion matrix for the YOLOv5x6 network. Each row of the confusion matrix represents the predicted variety; each column represents the true variety; and the background FN identifies the missed variety without detection frame and those judged as background. The confusion matrix displays every variety of barley detection accuracy and presents the confusing relation with each of the other barley seed varieties. Background FN precision was 0%, which means all seeds were located in the detection frame and detected without missing seeds in the test dataset. All barley seed recognition precisions were over 85%, and many were close to 100%; except for the Cele, which was 97.9%, and the Hock, which was 85.2%. The confusion matrix results proved that the well-trained YOLOv5x6 could detect all barley seeds and achieve high accuracy. It detects all nine barley seed varieties fast and accurately; while 3% of Cele was falsely detected as Lega, 7% of Hock was falsely detected as Lega, and 8% of Hock was falsely detected as Syne. Figure 16 shows that both Hock and Syne have around 3000 instances, with Lega having the most, with around 7000 instances, and Cele at least, with around 2500 instances. Fewer Hocks and Celes are falsely detected as Lega at a high rate. However, Hock was falsely detected as Syne at a high rate, and their numbers were similar. It proved that many factors affect the training results; not only the quantity, but also the color, size, posture, distribution, and even uncertain characteristics of the barley seeds. YOLOv5x6 trained the barley seed dataset with various complex identified features to achieve a well-trained model and high performance.

**Figure 18:**
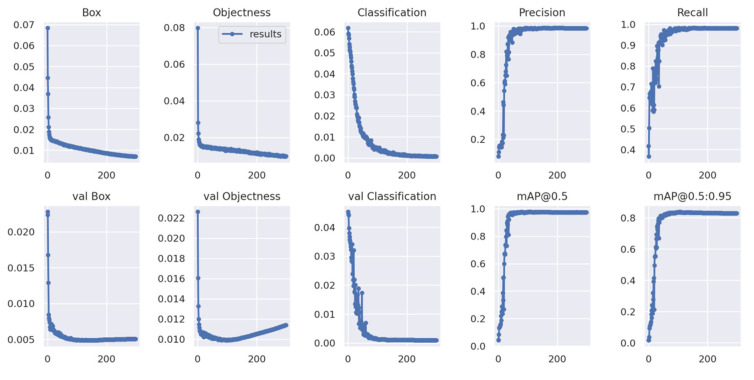
Training result plots of box loss, objectness loss, classification loss, precision, recall, and mean average precision (mAP) over the training epochs for the training and validation datasets.

Figure 18 displays detection result plots of barley seeds after 300 epochs of training. The box loss plot shows the bounding box loss of barley seeds; the objectness loss plot shows the average detection loss value of barley seeds; the classification loss plot shows the average classification loss value of barley seeds. The smaller the loss values of the box, objectness, and classification loss, the higher the accuracy of the barley seeds training dataset. In contrast, the val box loss plot, val objectness loss plot, and val classification loss plot are taken from the test dataset of barley seeds. The precision plot shows the ratio of true positive values to the total positive values of the barley seed datasets. The recall plot shows the percentage of true positive values to all the values in the relevant class from the barley seed dataset, which describes how many of the true positive seed samples in the test set were selected by the second classifier. The mAP@0.5 shows the average mAP with an IOU threshold greater than 0.5. The mAP@0.5:0.95 shows the average mAP value, while the different IOU thresholds were set as 0.5 to 0.95 with a step size of 0.05 [30]. The mAP@0.5:0.95 plot indicates barley detection efficiency with a different range of thresholds. When mAP values are bigger, the performance of barley seed detection is better. YOLOv5x6 displayed high performance for barley seed recognition.

**Figure 19:**
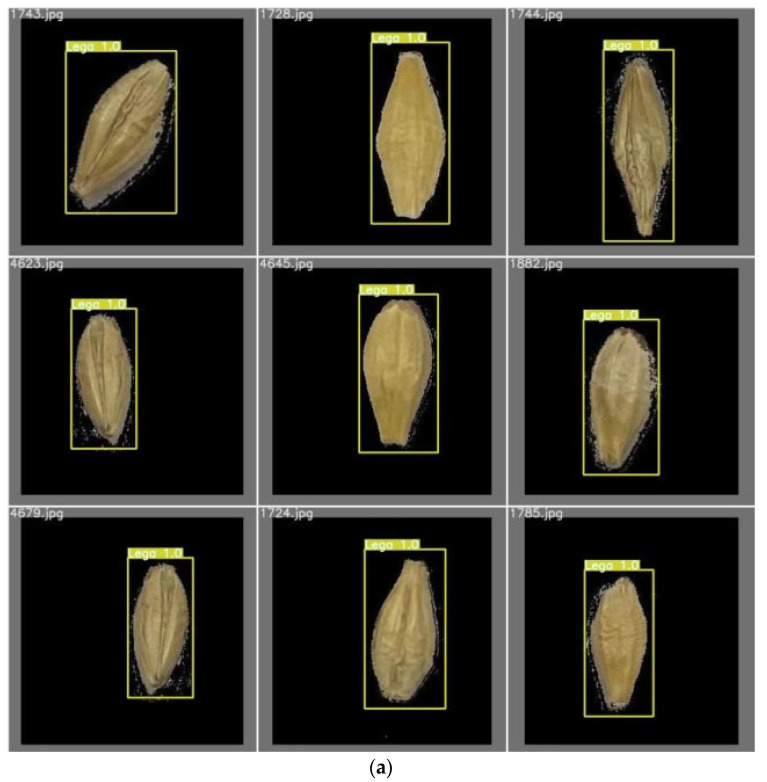

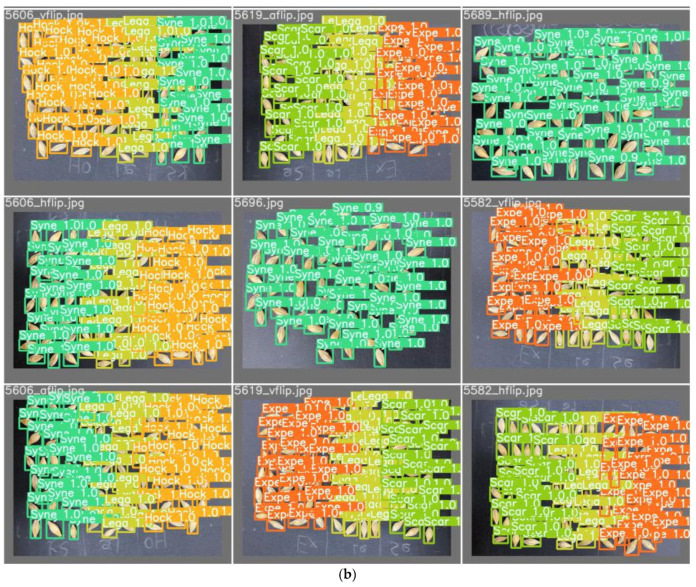
Some examples of barley seed detection results: (**a**) some single-barley seed identification images; (**b**) some multi-barley identification images.

Figure 19 displays some barley seed detection results, which proved to be effective. Figure 19a displays some single-barley seed identification accuracy close to 100%. Figure 19b displays some multi-barley identification accuracy, showing that the detection accuracy of these barley seeds was almost over 90% and close to 100%. Single-barley and multi-barley seed images were detected with high performance and without missed seeds. After the well-trained YOLOv5x6 model was built with nine categories of barley seeds, any iPhone 11 Pro captured high-resolution images with randomly placed barley seeds input well-trained models will achieve high detection results.

## 6. Conclusions

There has been no research conducted to automatically and efficiently detect nine varieties of barley seeds of random amounts and varieties in one image. This study established an automatic, quick, and reliable multi-barley seed detection method with high detection performance based on iPhone images and the YOLOv5x6 network, which can be widely used in malting and brewing industries for barley quality assessment. Evaluating barley seed variety is crucial for assuring the appropriate barley variety seeds are used to make premium malt and beer. A well-prepared dataset and improved training models were used to automatically detect barley seeds. The automatic barley seed detection method will completely replace the manual testing method, with higher detection speed, accuracy, and better robustness.

According to the different varieties of barley seed requirements, our project can adjust to different detailed functions. When more barley seed variety detection is required, barley seed images can be captured and the outlined data-segmentation algorithm and data-augmentation method can be used to acquire additional varieties of barley seed datasets. The well-adjusted hyperparameters of the YOLOv5x6 network training can be used to determine more varieties through barley seed detection. In the future, our research interests will focus on integrating the well-trained YOLOv5x6 model of barley seed detection into portable devices, such as smartphones, tablets, and other smart mobile devices. In the future, anyone could potentially take a photo of barley seeds to check the barley seed variety on smart mobile devices. Moreover, the ideal barley seed features are plump and sized grains; our research can also focus on intelligently identifying the features of size, color, and shape to select close to ideal barley seeds or assess the quality of barley seeds for the farming, malting, and brewing industries.

## Figures and Tables

**Table 1:** Training computer configuration list.

Item	Model
CPU + Motherboard	CPU Ryzen 9 Motherboard X570 AORUS Ultra
CPU Cooler	Noctua NH-U14S TR4-SP3 82.52 CFM CPU Cooler
Memory	Corsair Vengeance 64 GB
Storage	Samsung 1TB SSD
Video Card	RTX 3090

**Table 2:** YOLOv5 series trained model results list.

Model Category	Precision	Recall	MAP	Training Time
YOLOv5s	84.6%	78.4%	87.3%	6.68 h
YOLOv5m	83.7%	85.7%	90.5%	7.89 h
YOLOv5l	94.3%	94.8%	96.2%	10.72 h
**YOLOv5x6**	**98.4%**	**98.1%**	**97.5%**	**19.16 h**
YOLOv5x6 on multi-barley seed dataset	58.9%	71%	57.4%	7.68 h

## Data Availability

The datasets generated to obtain the results presented in this article are available from the corresponding authors upon reasonable request (wulushen@163.com).
